# First report on molecular docking analysis and drug resistance substitutions to approved HCV NS5A and NS5B inhibitors amongst Iranian patients

**DOI:** 10.1186/s12876-021-01988-y

**Published:** 2021-11-24

**Authors:** Zahra Hasanshahi, Ava Hashempour, Farzane Ghasabi, Javad Moayedi, Zahra Musavi, Behzad Dehghani, Heidar Sharafi, Hassan Joulaei

**Affiliations:** 1 Shiraz HIV/AIDS Research Center, Institute of Health, Shiraz University of Medical Sciences, Shiraz, Iran; 2 Baqiyatallah Research Center for Gastroenterology and Liver Diseases, Baqiyatallah University of Medical Sciences, Tehran, Iran; 3 Middle East Liver Diseases (MELD) Center, Tehran, Iran

**Keywords:** HCV, NS5A, NS5B, Drug-resistance, Bioinformatics, Iran

## Abstract

**Background:**

NS5A and NS5B proteins of hepatitis C virus (HCV) are the main targets of compounds that directly inhibit HCV infections. However, the emergence of resistance-associated substitutions (RASs) may cause substantial reductions in susceptibility to inhibitors.

**Methods:**

Viral load and genotyping were determined in eighty-seven naïve HCV-infected patients, and the amplified NS5A and NS5B regions were sequenced by Sanger sequencing. In addition, physicochemical properties, structural features, immune epitopes, and inhibitors-protein interactions of sequences were analyzed using several bioinformatics tools.

**Results:**

Several amino acid residue changes were found in NS5A and NS5B proteins; however, we did not find any mutations related to resistance to the treatment in NS5B. Different phosphorylation and few glycosylation sites were assessed. Disulfide bonds were identified in both proteins that had a significant effect on the function and structure of HCV proteins. Applying reliable software to predict B-cell epitopes, 3 and 5 regions were found for NS5A and NS5B, respectively, representing a considerable potential to induce the humoral immune system. Docking analysis determined amino acids involved in the interaction of inhibitors and mentioned proteins may not decrease the drug efficiency.

**Conclusions:**

Strong interactions between inhibitors, NS5A and NS5B proteins and the lack of efficient drug resistance mutations in the analyzed sequences may confirm the remarkable ability of NS5A and NS5B inhibitors to control HCV infection amongst Iranian patients. The results of bioinformatics analysis could unveil all features of both proteins, which can be beneficial for further investigations on HCV drug resistance and designing novel vaccines.

**Supplementary Information:**

The online version contains supplementary material available at 10.1186/s12876-021-01988-y.

## Background

Hepatitis C virus (HCV) is the principal cause of chronic hepatitis, cirrhosis, and hepatocellular carcinoma (HCC), resulting in hepatic associated-disease. The HCV genome is responsible for encoding structural (C, E1, and E2) and nonstructural (p7, NS2, NS3, NS4A, NS4B, NS5A, and NS5B) proteins [1]. NS5A has an N-terminal amphipathic alpha-helix (amino acids 5–25) and three structural domains [2]. The first domain contains Zn2 + -binding and RNA-binding motifs that are vital for HCV replication [3]. Domain two of NS5A controls RNA replication and domain three is essential for virus assembly. NS5B contains a hydrophobic region at the C-terminus responsible for membrane anchorage. NS5B is essential for HCV replication and is subject to considerable purifying selection to maintain the critical functions [2–4].

Interferon (IFN) was an effective therapeutic agent for HCV infection [5, 6]; however, its efficacy even with ribavirin (RBV) is not more than 50%, especially for patients infected with genotype 1 [7]. Direct-acting antivirals (DAAs) are novel antiviral reagents that directly target HCV viral proteins with high antiviral effects, resulting in an acceptable virological response rate [8]. NS5A and NS5B polymerase inhibitors, with and without combination with peg-interferon (peg-IFN) and RBV were recently introduced [9]. Approved DAAs such as protease inhibitors, NS5A inhibitors, and polymerase inhibitors are available for clinical usage.

Daclatasvir (DCV) is a first-in-class HCV NS5A inhibitor with potency and broad genotype coverage in vitro*,* and ever since several NS5A inhibitors have been introduced [2]. Although NS5A inhibitors show high potential among all DAAs, the main problem with the use of NS5A inhibitors is the emergence of resistance-associated substitutions (RASs). Several amino acid changes in NS5A were associated with various resistance levels to NS5A inhibitors [10]. In addition, NS5B is one of the suitable drug targets that inhibit HCV directly, and recently, the FDA approved two NS5B inhibitors, sofosbuvir, and dasabuvir [11]. Previous studies have shown the mutations that reduce susceptibility to DAA therapies can occur naturally during patients’ treatment.

During the last decade, bioinformatics analysis has played a significant role in defining the viral and bacterial mutations, protein features and designing novel vaccines [12, 13]. This research aimed to compare and identify drug-resistant mutations amongst Iranian patients infected with HCV genotypes 1a and 3a. Furthermore, we used several reliable software to investigate the features of NS5A and NS5B proteins, the effects of mutations on the function of these regions, physicochemical properties, B-cell epitopes, as well as secondary and tertiary structures. In addition, the impact of NS5A and NS5B mutations on the drug efficacy was evaluated by docking analysis.

## Methods

### Patients

A total of 87 patients with chronic hepatitis C (CHC) genotype 1a and 3a who were naïve for the treatment of HCV infection with negative antibody response against HIV and HBsAg enrolled at Shariati Hospital, Tehran University of Medical Sciences, Iran.

### Determination of HCV viral load

Viral load of samples was done by qualitative RT-PCR assay (Roche COBAS Amplicor HCV Monitor v 2.0, Roche Diagnostics, Mannheim, Germany) with a detection range of 12–100,000,000 IU/mL.

### HCV molecular genotyping

According to the manufacture’s instruction of INNO-LiPA HCV II kit (Innogenetics, Ghent, Belgium), Genotyping of HCV was performed.

### HCV sequence alignment and primer design

Complete genome sequences of HCV-1 and HCV-3a genotypes from different geographical regions were retrieved from the Los Alamos HCV sequence database. Multiple sequence alignment was performed, using CLC sequence viewer, and the consensus sequence was used to design primers for the NS5A and NS5B regions in both HCV genotypes 1a and 3a. Newly designed primers are listed in Table 1.

**Table 1:** Primers used for nested-PCR assay

Primers		Sequences
NS5A 1a	F1	5-TACGTGCCGGAGAGCGATG-3
	R1	5-AAGTTGCCTTGAGAGATGGAGC-3
	F2	5-CCTCACTGTRACCCAGCTCC-3
	R2	5-AGGAGCTGGCCACAGAAGG -3
NS5A 3a	F1	5-CACCGACGCACTATGTTCC-3
	R1	5-CAAGTGGCCTTCAACGACG-3
	F2	5-GAGGGTCACTGCATTGCTG-3
	R2	5-AGTTGGCTGGCGGATGAG-3
NS5B 1a	F1	5-TGGCCATCAAGTCCCTCAC-3
	R1	5-CGCTATTGGAGTGAGTTTGAG-3
	F2	5-GGCCCTCTTACCAATTCAAG-3
	R2	5-TGAAGAGGTACTTGCCACATATG-3
NS5B 3a	F1	5-GAAGAGGAGATATACCAATGCTG-3
	R1	5-CGTGACACGCTGTGATAAATG-3
	F2	5-ACGGAGCGGCTTTACTGC-3
	R2	5-CGTACCGCCCAATTAAAGAG-3

### HCV RNA extraction, cDNA preparation and nested-PCR

According to the manufacturer’s instructions commercial kit (Invitec, Germany), viral RNA was extracted from 200 μL of the serum and eluted in 100 μL of elution buffer. The extracted RNA was subjected to cDNA synthesis using a random primer and M-MLV reverse transcriptase. Reverse transcription was performed at 25 °C for 10 min, followed by 42 °C for 90 min, and 72 °C for 10 min. NS5A and NS5B regions were amplified by nested-PCR in a 25 μL final volume PCR reaction contained 1 × PCR buffer, 1 mM of dNTP, and 0.2 µM of each primer.

In both steps of nested-PCR, amplification consisted of 35 cycles of denaturation at 94 °C for 45 s, annealing at 54 °C for 45 s, and extension at 72 °C for 45 s, followed by a final extension at 72 °C for 8 min. The positive amplicons were purified, using a commercial gel extraction kit (QiagenGmbH, Hilden, Germany), followed by Sanger sequencing with the limit of detection ~ 15–20%.

### Amino acid changes

Substitutions with respect to the reference sequences (accession numbers for 1a reference sequence were AF009606 and for 3a it was D17763) were confirmed by CLC sequence viewer version Beta (QIAGEN).

### Primary sequence analysis

"Expasy’sProtParam” (http://expasy.org/tools/protparam.html) was employed to determine the theoretical isoelectric point (pI), molecular weight, extinction coefficient, instability index, aliphatic index, and grand average hydropathy (GRAVY) [14, 15].

### Functional characterization

Serine, threonine, and tyrosine phosphorylation sites prediction was carried out by DISPHOS (http://www.dabi.temple.edu/disphos/pred.html) [16] and NetPhos (http://www.cbs.dtu.dk/services/NetPhos/) [17]. N-glycosylation sites were predicted using NetNGlyc (http://www.cbs.dtu.dk/services/NetNGlyc/) and GlycoEP (http://www.imtech.res.in/raghava/glycoep/submit.html) [18]. In addition, Dianna (http://clavius.bc.edu/~clotelab/DiANNA/) [19] was employed to define Cysteine state and Disulfide Bond.

### B-cell and T-cell epitopes prediction

www.immuneepitope.org (http://tools.immuneepitope.org/tools/bcell/iedb_input), ABCpred software (http://www.imtech.res.in/raghava/abcpred/) [20], and BcePred (http://www.imtech.res.in/raghava/bcepred) [21] were used to carry out B-cell epitope prediction. ProPred-I (http://www.imtech.res.in/raghava/propred1/), propred (http://www.imtech.res.in/raghava/propred/), and Immune Epitope Database (IEDB) (http://tools.immuneepitope.org/main/) were employed to predict T-cell prediction.

### Secondary and tertiary structures prediction

SOPMA software was applied to calculate the secondary structure at (http://npsa-pbil.ibcp.fr/cgi-bin/npsa_automat.pl?page=npsa_sopma.html) [22] and the results were confirmed by Phyre server at (http://www.sbg.bio.ic.ac.uk/phyre) [23]. "I-TASSER" at (http://zhanglab.ccmb.med.umich.edu/I-TASSER) [24, 25] was employed to construct the tertiary structure of the selected sequences. 3D models were refined, using GalaxyRefine (http://galaxy.seoklab.org/cgi-bin/submit.cgi?type=REFINE) [26]. To find the best model, "Qmean" at (http://swissmodel.expasy.org/qmean/cgi/index.cgi) [27]," Rampage "at (http://mordred.bioc.cam.ac.uk/~rapper/rampage.php) [28], ERRAT at (https://servicesn.mbi.ucla.edu/ERRAT/), and ProSA-web (https://prosa.services.came.sbg.ac.at/prosa.php) were used.

### Docking analysis

Docking with four NS5A inhibitors (Daclatasvir, Elbasvir, Ledipasvir, and Ombitasvir) and NS5A protein as well as NS5B and 2 approved NS5B inhibitors (Dasabuvir and Sofosbuvir**)** was done using Hex and PatchDock servers. The parameters used for the Hex docking process were: Correation type—Shape only, FFT Mode—3D fast lite, Grid Dimension—0.6, Receptor range—180, Ligand Range—180, Twist range—360, and Distance Range—40. The parameters used for the PatchDock docking process were: Clustering RMSD: 1.5, Complex Type: Protein-small ligand.

### Statistical analysis

All statistical analyses were performed, using the statistical package for the social sciences (SPSS version 15, USA). We used the Shapiro–Wilk test to assess normal distribution, the Pearson Chi-square test for categorical data, and ANOVA for comparison of the mean values. A p-value of less than 0.05 was considered to be statistically significant.

## Results

### Patients

The majority of patients were male (65.7%) and 50.4% of them were under the age of 40 years old. Higher viral load defined as HCV-RNA levels ≥ 400,000 IU/mL detected in 66.1% of samples which was not statistically related to age, gender, and viral load.

### Amino acid changes analysis

Comparison of the patients’ sequences and isolated reference showed several amino acid residue changes, shown in Table 2.

**Table 2:** List of amino acid substations found in this study

Mutation	NS5A 1a (20)	Mutation	NS5A 3a (21)	NS5B 1a (15)	Mutation	NS5B 3a (31)	Mutation
R 44 K	14 (70%)	T 7 N	1 (4.7%)	N 444 D	12 (80%)	N 317 G	27 (87%)
V 75 A	8 (40%)	A 17 S	8 (38%)	R 300 Q	12 (80%)	A 366 P	31 (100%)
R 78 K	14 (70%)	A 21 T	10 (47%)	Q 309 R	6 (40%)	K 389 R	18 (58%)
K 107 T	20 (100%)	A 62 S	6 (28.57%)	A 327 Q	9 (60%)	I 448 V/T	21 (67.7%)
I 121 V	8 (40%)	T 64 S	4(19%)	H 374 Y	2 (13.3%)	A 398 P	4 (12.9%)
R 123 Q	14 (70%)	H 85 Y	4 (19%)	S 431 G	2 (13.3%)	M 436 L	4 (12.9%)
S 131 T	20(100%)	S 103 P	6 (28.57%)	V432 I	2 (13.3%)	D 386 N	12 (38.7%)
I 144 V	20 (100%)	N116 S	5 (23.8%)	I 434 V	1 (6.6%)	K 314 R	9 (29%)
E 171 D	12 (60%)	E 137 G	4 (19%)	P 461 L	1 (6.6%)	R 384 L	9 (29%)
S 174 T	8 (40%)	E 137 G	4 (19%)	S 335 N	1 (6.6%)	P 275 L	2 (6.45%)
E 181 D	15 (75%)	D 172 E	8 (38%)			A 315 V	1 (3.22)
L 199 V	8 (40%)	M 176 T	6 (28.57%)			T 350 A	1 (3.22)
		H 180 N	9 (42.85%)			A 347 T	2 (6.45%)
		T 183 A	7 (33.33%)			K 314 R	2 (6.45%)
		Q 309 R	6 (40%)			K 387 E	1 (3.22%)
		A 327 Q	9(60%)			D 392 E	1 (3.22%)
						Y 391 C	1 (3.22%)
						T 310 M	1 (3.22%)
						R 343 K	1 (3.22%)
						A 348 V	1 (3.22%)
						A 403 T	1 (3.22%)
						V 490 M	1 (3.22%)

For NS5A sequences, the most prevalent mutations in 1a genotype were K 107 T (100%), S 131 T (100%), and I 144 V **(**100%), and for 3a genotypes, the mutations were A 21 T (47%), H 180 N (42%) and T 183 A (33.3%). The most prevalent mutations in NS5B 1a and 3a were Q 300 R, and D 444 N and N 317 G, A 366 P, I 448 V/T, respectively.

### 3-5-Protparam results

PI analysis results showed that both NS5A and NS5B proteins are basic. The results of both proteins showed the appropriate stability in yeast and *E.coli*; however, “protparam” analysis predicted that they are not stable in mammalian cells. The instability index, an estimate of the stability of a protein in a test tube, showed both proteins to be unstable. Aliphatic index, a positive factor for the increase of thermostability of proteins, indicated that both proteins are the thermostable proteins. In addition, GRAVY is a Hydropathicity index and a negative score indicates NS5A and NS5B are hydrophilic. Moreover, the results suggested insignificat differences between 1 and 3a genotypes (Table 3).

**Table 3:** Estimation of NS5A and NS5B properties by “protparam”

PROTPARAM	NS5A 1a	NS5A 3a	NS5B 1a	NS5B 3a
Number of amino acids	229	233	591	591
Molecular weight	25,637.51	25,565.16	65,311.27	66,134.92
Theoretical pI	8.34	7.53	9.12	9.02
Estimated half-life	1.9 h (mammalian reticulocytes, in vitro)	1.9 h (mammalian reticulocytes, in vitro)	1.9 h (mammalian reticulocytes, in vitro)	1.9 h (mammalian reticulocytes, in vitro)
	> 20 h (yeast, in vivo)	> 20 h (yeast, in vivo)	> 20 h (yeast, in vivo)	> 20 h (yeast, in vivo)
	> 10 h (Escherichia coli, in vivo)	> 10 h (Escherichia coli, in vivo)	> 10 h (Escherichia coli, in vivo)	> 10 h (Escherichia coli, in vivo)
Instability index	Unstable 45.71	Unstable 44.48	Unstable 41.41	Unstable 45.66
Aliphatic index	70.66	69.53	86.7	85.37
GRAVY	− 0.262	− 0.226	− 0.103	− 0.219

Almost both genotypes showed similar features. However, there were significant differences between pI of NS5S 1a and 3a genotypes

### Post-translational modification analysis

Post-translational modification (PTM) prediction of both proteins is summarized in Table 4. Prediction of serine, threonine, and tyrosine phosphorylation sites identified 14 positions in NS5A 1a genotype and 10 residues in N55A 3a genotype, amongst which 6 positions were common in both genotypes. In N55B protein, 9 and 10 positions were found in 1a and 3a genotype, respectively, while no similarity was found between the two genotypes. Using "NetNGlyc" and "GlycoEP, the glycosylation outcomes of NS5A and NS5B proteins revealed merely one site for NS5A 1a (69) and 3 sites for NS5A 3a (69,103,105). In addition, amino acid 369 was considered as a possible glycosylation site for NS5B 1a and 2 positions, 379 and 545, were predicted for NS5B 3a. Disulfide bonds prediction showed several potential bonds.

**Table 4:** Prediction of post modification sites for both N55A and N55B proteins of two genotypes of 1a and 3a

Genotypes	Phosphorylation
N55A 1a	99 T, 103 S, 106 Y, 117 S, 118 Y, 129 Y, 134 T, 181 S, 201 S, 207 S, 222 S, 225 S, 229 S, 232 S
N55A 3a	99 T, 114 S,118 Y, 129 Y,131 S,146 S,200 T,207 S, 222 S,225 S
N55B 1a	64 Y, 103 Y, 335 S, 346 Y, 347 S, 399 T, 506 S, 513 S, 561 Y
N55B 3a	34 S, 56 S, 106 S, 113 Y, 114 S, 122 S, 142 T, 357 S, 395 T, 409 T

Genotypes	Glycosylation
NS5A 1a	69
NS5A 3a	69,103,105
NS5B 1a	369
NS5B 3a	379, 545

Genotypes	Disulfide bonds
NS5A 1a	39–59, 39–140, 39–142, 39–190, 98 –165
NS5A 3a	39–57, 39–59, 57–140, 101–165, 142–190
NS5B 1a	14–46, 146–170, 243–279, 242–274, 324–366, 521–575
NS5B 3a	9–156, 24–99, 233–253, 252–284, 272–305, 326–376, 504–531

Results showed several phosphorylation and glycosylation sites as well as disulfide bonds in both proteins

### B-cell epitopes prediction

The combination of all prediction samples showed 6, 4, 2, and 3 as the potentials B-cell epitopes in NS5A 1a, NS5A 3a, NS5B 1a, and NS5B 3a, respectively (Table 5).

**Table 5:** B-cell epitopes of NS5A and NS5B proteins for 1a and 3a genotypes

Genotypes	B-cell epitopes
NS5A 1a	50–85, 163–175, 92–103, 180–197, 126–151, 215–225
NS5A 3a	90–110, 128–142, 215–233, 185–197
NS5B 1a	204–231, 342–400
NS5B 3a	20–34, 160–190, 230–260

There was no similarity between the two genotypes

### Secondary and tertiary structures prediction

**Fig. 1:**
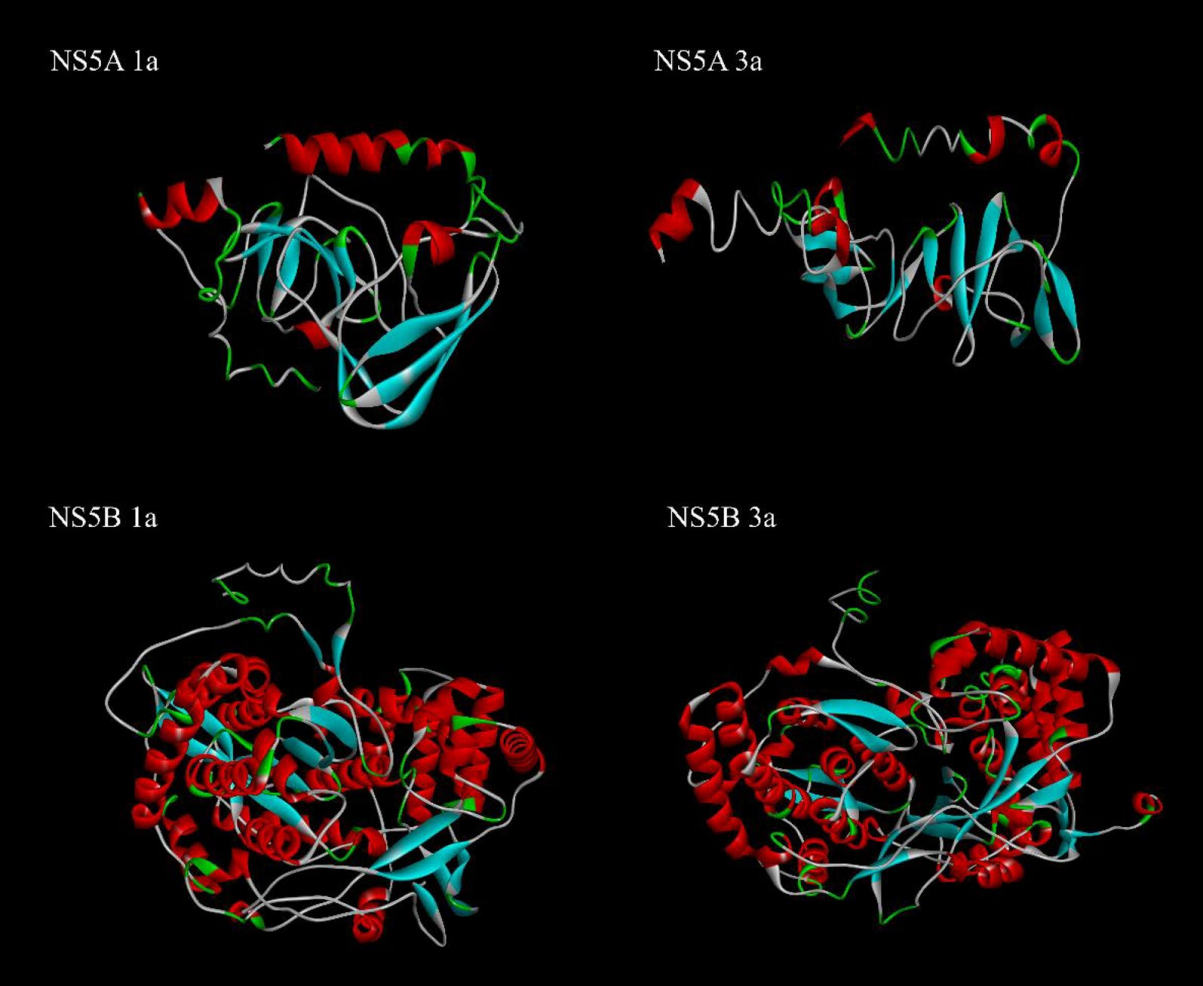
Final refined tertiary structures for NS5A and NS5B proteins by I-TASSER and evaluated by 4 online programs. Results indicated significant differences between 1 and 3a genotypes

Secondary structure prediction by SOPMA software is summarized in Table 6. SOMPA showed by far, the majority of NS5A protein contained Random coil in both 1a and 3a genotypes; however, in NS5B, the majority was Alpha helix (43%) and then Random coil (36%). The evaluated results of the suggested 3D models are summarized in Table 7, and selected models are bolded. The best "I-TASSER" tertiary structures are illustrated in Fig. 1.

**Table 6:** Analysis of the secondary structure of NS5A and NS5B, 1a and 3a genotypes

Sopma	NS5A 1a	NS5A 3a	NS5B 1a	NS5B 3a
Alpha helix (Hh)	59 (25.76%)	61 (26.18%)	256 (43.32%)	258 (43.58%)
Extended strand (Ee)	45 (19.65%)	48 (20.60%)	78 (13.20%)	81 (13.68%)
Beta turn (Tt)	23 (10.04%)	25 (10.73%)	36 (6.09%)	39 (6.59%)
Random coil (Cc)	102 (44.54%)	99 (42.49%)	221 (37.39%)	214 (36.15%)

The majority of secondary structures in NS5A and NS5B were random coil and Alpha helix, respectively

**Table 7:** The evaluation of refined models by 4 onlien programs

HCV	Models	Qmean	ERRAT	ProSA-web	Rampage (Ramachandran plot)
NS5A 1a	1	− 0.89	72.8507	− 4.72	94.3%, 3.5%
	**2**	− **0.45**	**69.6833**	− **4.58**	**93.8%, 3.5%**
	3	− 1.12	68.1818	− 4.49	93.8%, 4.0%
	4	− 0.9	69.6833	− 4.7	93.8%, 3.5%
	5	− 0.59	70.5882	− 4.75	93.8%, 4.4%
	Itasser	− 4.64	78.733	NA	85.9%, 8.4%
NS5A 3a	1	− 4.45	85.3333	− 4.48	88.7%, 6.5%
	**2**	− **4.09**	**85.7143**	− **4.55**	**89.2%, 6.5%**
	3	− 4.46	87.0536	− 4.58	88.7%, 6.9%
	4	− 3.98	80.8889	− 4.43	88.7%, 6.9%
	5	− 4.34	86.6667	− 4.53	89.2%, 6.5%
	Itasser	− 6.56	86.2222	na	73.2%, 18.2%
NS5B 1a	1	− 1.06	83.737	− 10.85	96.3%, 3.2%
	2	− 1.14	88	− 10.85	96.1%, 3.2%
	3	− 1.19	89.4464	− 10.79	95.6%, 4.1%
	**4**	− **1.16**	**85.4167**	− **10.71**	**96.1%, 3.2%**
	5	− 1.10	86.8284	− 10.82	95.6%, 3.7%
	Itasser	− 4.17	93.1389	na	87.3%, 8.7%
NS5B 3a	1	− 1.26	86.7241	− 11.59	96.6%, 2.9%
	**2**	− **1.06**	**92.6117**	− **11.35**	**96.6%, 3.1%**
	3	− 1.37	91.7241	− 11.49	96.6%,2.9%
	4	− 1.25	93.1271	− 11.38	96.%, 2.7%
	5	− 1.11	92.4138	− 11.23	96.8%,2.7%
	Itasser	− 4.29	94.3493	na	87.1%, 9.3%

The final selcetd models are bolded

### Docking results

Additional file 1: Figs. S1–S12 and Tables 8, 9, and 10 show the docking results between NS5A, NS5B, and drug inhibitors.

**Table 8:** The Hex docking analysis between NS5A and NS5B proteins and the approved HCV inhibitors

Proteins	HCV inhibitors					
	Daclatasvir	Elbasvir	Ledipasvir	Ombitasvir	Dasabuvir	Sofosbuvir
NS5A 1a	− 313	− 318	− 352	− 330		
NS5A 1a (Mutant GLU181)	_	− 150	_	_		
NS5A 3a	− 222	− 194	− 222	− 204		
NS5B 1a					− 254	− 282
NS5B 3a					− 224	− 234

**Table 9:** The PatchDock docking analysis between NS5A and NS5B proteins and the approved HCV inhibitors (according atomic contact energy)

Proteins	HCV inhibitors					
	Daclatasvir	Elbasvir	Ledipasvir	Ombitasvir	Dasabuvir	Sofosbuvir
NS5A 1a	− 153.79	− 395.54	− 415.40	− 455.13		
NS5A 1a (Mutant GLU181)	–	− 114.63	–	–		
NS5A 3a	− 306.60	− 91.09	− 298.97	− 460.31		
NS5B 1a					− 178.73	− 250.39
NS5B 3a					− 101.30	− 250.39

**Table 10:** The list of amino acid residues involved in the interactions between NS5A, NS5B, and HCV inhibitors

Proteins	HCV inhibitors					
	Daclatasvir	Elbasvir	Ledipasvir	Ombitasvir	Dasabuvir	Sofosbuvir
NS5A 1a	TRP9 PRO35 PHE36 ARG78 TRP84	GLU181 TYR129 SER186 GLY185	PRO192 GLU193 TYR129 THR210 THR213 ALA214	HIS150 TYR182 GLN187 TYR129 PRO194 PHE127 ARG204		
NS5A 1a (Mutant GLU181)	–	GLU327 ASP292	–	–		
NS5A 3a	TRP9 PRO35 PHE36 ARG78 TRP84	GLU181 TYR129 SER186 GLY185	PRO192 GLU193 TYR129 ARG122 THR210 THR213 ALA214	HIS150 TYR182 GLN187 TYR129 PRO194 PHE127 ARG204		
NS5B 1a					GLU202 ARG591 ARG465 LEU545 LEU547	HIS467 GLY468 ARG591 ARG465 LEU547
NS5B 3a					MET217 LYS216 ARG213 PRO199 GLU212 GLN209 TYR205	ALA26 ARG411 HIS412 PRO414 HIS105 PRO104

## Discussion

For many years, a combination of pegylated interferon-α (PEG-IFN)-α and ribavirin was the standard therapy for chronic hepatitis C patients as an indirect antiviral treatment without any direct target on HCV protein or RNA element. In 2016, the Clinical Management of Hepatitis C in Iran, prepared by Iran Hepatitis Network (IHN) recommended Daclatasvir, Sofosbuvir, Ledipasvir, and Ribavirin for the treatment of HCV-infected patients [29].

In the present study, our data showed that NS5B and NS5A inhibitors may be beneficial to prescribe for Iranian HCV-infected patients. Docking analysis suggested the high energy values between NS5A and NS5B inhibitors and the 3D models of NS5 proteins. The higher docking score represented better binding ability indicated the strong attachment of inhibitors to NS5A and NS5B proteins to suppress viral functions that would be contributed to the promising treatment outcome.

The highest energy values belonged to NS5A 1a with selected NS5A inhibitors; however, NS5B results did not show significant differences between 1 and 3a genotypes and NS5B inhibitors. The amino acids have located in the attachment region of NS5B and NS5B inhibitors showed different patterns in two genotypes; but, there was no mutation in the mentioned regions. The amino acids in the attachment regions of NS5A and NS5B proteins not only were highly conserved in genotypes 1a and 3a, but also they were preserved in all samples. These conserved regions provide an absolute opportunity for drug to target the viral protein easily that causes increased sensitivity to NS5 inhibitors leading to a favorable clinical treatment. There was only one substitution (E181D) in the attachment region (NS5A-1a and Elbasivir) with a frequency of 75% in NS5A 1a sequences. The substitution E181D in NS5A 1a did not affect the other NS5A inhibitors, but Elbasvir could significantly change the energy value. The presence of this mutation could result in deletion in a phosphorylation site; therefore, suggested NS5A inhibitors are functional in controlling HCV infection amongst Iranian patients.

Compared with previous studies, three new mutations (62, 309, and 327) were identified in NS5A sequences, which might have drug resistance effects. From 2014 to 2016, Grandal et al. [30] conducted a cohort study in Spain in which 232 patients naïve to NS5A inhibitors were enrolled. They have reported 5 resistance-associated substitutions (RASs) mutations (M28A/G/T, Q30D/E/H/G/K/L/R, L31M/V/F, H58D, and Y93C/H/N/S), while in our study we did not find any of these mutations. This discrepancy may be due to this situation that a high proportion of patients harbored at least one of the factors related to poor response including cirrhosis status, being interferon experienced, and HCV-RNA > 800,000 IU/mL, that can have a detrimental effect on the emergence of the new strains with higher pathogenicity. Contrary to a study by Aldunate et al. [31], we did not find any of the mutations introduced in their study. They found 3 mutations (L31 V/M, H58P, and K24Q) in NS5A sequences and 4 substitutions (A421V, C451R, Q556R, and A553G) in NS5B region. The disparity between the results of the present study and Aldunate et al. [31] might be explained from the view that the latter evaluated only 31 patients from South America that is geographically far from the Middle East.

From 2015 to 2018, a large-scale RASs study was conducted with 878 Chinese patient’s samples from 27 provinces across the country who were infected with five HCV genotypes: 1b, 2a, 3a, 3b, and 6a. Although all patients were DAA-naïve treatment, fifty-two percent of patients had documentation of experiencing interferon-based therapy at sample collection. We did not find any substitution suggested by Lu et al. [32], describing 3 high prevalence mutations (Y93H, A30K, and L31M) in NS5A samples. Other clinical features of patients were not included in the study, but individuals with various genetic backgrounds were evaluated from diverse regions of China [32]. Also, Ramezani et al. [33] studied 209 DAA-naïve chronic HCV patients including 104 HCV mono-infected and 105 HCV/HIV co-infected registered in one of the hospitals in Tehran from May 2017 to February 2018. They found L28M, M28V, Q30H, and Y93H/N substitutions in NS5A and V321A/I, C316Y, S282R, and L159F substitutions in NS5B, but these substitutions were not detected in the present study. The difference between our data with previous study might be due to different sample sizes, patient populations, diverse geographical regions, diversity between isolates’ geographic region, and the genetic background of the host. For this reason, we believe that there is a need to contribute data from our region, for which there is not have enough information.

In a large cohort study on Italian patients, Bertoli et al. [34] investigated 1445 HCV-infected DAA-naïve individuals from 23 Italian clinical centers between 2011 and 2016. More than 50% of patients suffer from liver cirrhosis and less than 10% of them had a history of liver transplant or HCC. They did not find any significant associations among absence or presence of cirrhosis and prevalence of mutations. Overall, there was no similarity in NS5B substitutions between Bertoli’s report [34] and the present study. In the investigation by Stefano et al. [35], fifty HCV-infected Italian patients who experienced virological failure to a DAA-containing regimen were studied between January 2016 and July 2018. They found various RASs related to NS5A (Q30R, Y93C, L3lM, M28T, L31V, L28M, L31M, L31I, P58S, and P58S) significantly and NS5B (S556G and C451N) non-significantly. Moreover, sequence analysis of the NS5A and NS5B regions illustrated a significant correlation between the emergence of RASs and advanced fibrosis/cirrhosis and viral genotype, but not with viral load, sex, and age. In another cohort study of Stefano et al. [36], they characterized the genetic subtypes of HCV genotype 4 and identify the emergence of natural RASs in Saudi Arabia patients. All 17 patients attending the Hepatology Clinic at the Gastroenterology section at the King Fahad Hospital of the University, Al Khobar, Saudi Arabia, were DAA-naïve. Then they have treated with a daclatasvir plus sofosbuvir plus regimen for 12 weeks. Although we found natural RASs were found they were not associated with DAA failure was not observed. A sustained virological response (SVR) was obtained in all, but one who experienced a relapse. In three patients, NS5A region harbored clinically relevant RAS [L28M, L30R, and (L28M + M31L)] that were completely different from the RAS in the present study. In addition, several polymorphisms were found in NS5B region that neither associated with DAA resistance nor with apparent clinical impact. However, no mutations associated with resistance were observed among NS5B sequences. The discrepancy between Stefano’s studies results with the present report may be contributed to the different factors including sample size, geographic region, genotype, viral load, clinical situation, and age. NS5A and NS5B resistance data are still rare due to the high cost and late approval of DAAs, limited application caused by the inadequate management of public healthcare systems, the limited access of low-income or uninsured patients to healthcare providers, and the lack of enough and accurate clinical information of patients. It might be beneficial to optimize NS5-based treatment avoiding ribavirin‐related toxicities, and shortening therapy duration in the majority of HCV-infected patients.

The data of ProtParam determined the notable similarity in NS5A and NS5B amino acids in both 1a and 3a genotypes. But, the pI in NS5A showed a difference between the two genotypes which can be described by the diversity in the number of basic amino acids. Accurate prediction of the pI of viruses is beneficial for physical/chemical treatment processes and modeling virus behavior in environment [37].

In recent years, HCV-NS5A and NS5B recombinant proteins used for different approaches including vaccine development, HCV genotyping, serological diagnostic methods, and therapeutic applications [38–44]. There is not a general expression host system to act optimally for all mentioned purposes [45]; thus, several expression host systems should be considered for each object. Here, our findings indicated E.coli and yeast were the best available and appropriate expression systems for serological diagnostic methods and vaccine development, respectively. In other words, we were able to determine that yeast and E.coli could be appropriate hosts to express NS5A and NS5B proteins for the detection of anti-HCV antibody since they can tolerate high temperatures. In agreement with our prediction, various studies including Anwar et al. [46], Dou et al. [47], Kalamvoki et al. [48], and Alaee et al. [49] showed the high stability of NS5A protein in E.coli and confirmed this host could be a suitable host for NS5A expression. Moreover, the other host that provides all protein post-modification processes is yeast; therefore, NS5A and NS5B can be highly and appropriately expressed in such host to induce more appropriate features, even immunologic activity as a vaccine construct.

Although our prediction showed that NS5A and NS5B proteins are unstable in mammalian cells for less than 2 h, Huang et al. [50] and Polyak et al. [51] used Huh-7 and HeLa cells, respectively, to express NSBA, which showed the stability of this protein in both cell lines. In addition, Shimakami et al. [52] and Goh et al. [53] investigated the expression of NS5B in Huh7 and Huh7-DMB cell lines. The contrast between experimental and in silico studies can contribute to several factors that play a pivotal role in vivo that are not taken into consideration in the ProtParam software.

The NS5A is a phosphorylated protein has been shown to be involved mostly in HCV pathogenesis, oncogenic transformation, and resistance to IFN therapy [54]. The NS5B protein is a RNA dependent RNA polymerase (RdRp) and helps in the replication of HCV and have a role in HCC development [55].

PTMs of proteins involve the attachment of small proteins or functional groups such as glycosylation, phosphorylation, disulfide bridging, etc. to specific amino acids within the proteins that caused altering the structure and charge or hydrophobicity of proteins which are crucial for the proteins’ functioning. During viral infection, PTMs leads to increase protein solubilization, enhance protein antigenicity and virulence properties, viral replication, interferon response inhibition that play a crucial role in viral pathogenesis. In other words, host cells remove PMTs from viral proteins to inhibit viral protein synthesis, activate immune response pathways and control the virus replication to eliminate the virus by inactivating the viral proteins. Hopefully, bioinformatics and biochemaical analysis pave the way for development of new drugs to pharmacological suppress HCV- NS5A and NS5B proteins that may result in the restoration of strong immune responses, suppression of tumorigenesis that aim to eliminate the virus [56].

In addition, previous studies showed that NS5A phosphoprotein can disrupt the host interferon-induced antiviral response. Recently, it was suggested that phosphoprotein may take part in regulating HCV RNA replication, modulate host intracellular signaling pathways, and virion assembly. The phosphorylation process is highly regulated and cellular protein kinases are responsible for this process, which are suggested as the new target for antiviral therapeutic intervention [57]. Our analysis showed several phosphorylation sites in NS5A and determined high similarity between the two genotypes. Consistent with our outcome, Chong et al. [58] found some phosphoprotein sites and interestingly serine 222 was a common site between the two studies. While the functions of many phosphorylation sites in NS5A remains unclear, Chong et al. [58] identified three phosphorylation sites (serine 222, 235, and 238) in the NS5A using phosphoproteomics that played a critical role in replication, and Ser-235 dominated the Ser-222 and Ser-238 in HCV replication.

In line with the present data, previous studies showed alanine mutations in serine 225 led to NS5A hyper-phosphorylation reduction and replication of HCV genotype 2 [4, 58, 59]. In addition, Goonawardane et al. [60] focused on serine 225 (S225) and determined the contribution of S225 phosphorylation in the regulation of genome replication, interactions of NS5A with several host proteins, and the sub-cellular localization of NS5A. Mutations can affect the phosphorylation sites and our data suggested mutation in amino acid 131 could omit this phosphorylation site in 1a genotype. Furthermore, mutations in amino acids 103, 137, 181, and 183 could delete the different phosphorylation sites (103, 134, and 181) which might affect NS5A function in the infected cells.

While our analysis predicted several phosphorylation sites for NS5B, the phosphorylation pattern showed significant differences between the two genotypes. Han et al. [61] reported the vital role of NS5B in HCV replication and determined that the HCV NS5B phosphorylation has a positive regulatory function with different genotypes, virus strains, and different geographical regions. Our analysis determined mutation in amino acid 335 could omit this phosphorylation site in genotype 1a.

According to our result, predicted phosphorylation amino acids (S225, S222, and S235) in HCV NS5 amongst Iranian patients have a critical role in virus replication, pathogenesis, chronic infection, liver diseases, and antiviral therapeutic intervention [57]. Therefore, new treatment strategy that can inhibit NS5A and NS5B phosphorylation of vital amino acids can prevent HCV pathogenesis.

Understanding the process of NS5A and NS5B phosphorylation and its role in the HCV life cycle will not only reveal mechanisms of HCV-RNA replication and/or pathogenesis but may also provide new avenues for therapeutic intervention. There is some evidence showed NS5A induces liver pathogenesis, steatosis, and hepatocellular carcinoma that NS5A phosphorylation can affect these important functions directly or indirectly [62]. Furthermore, there are some reports suggested S225 phosphorylation disruption reduced the HCV replication [60] and virus assembly [16, 47, 59]. NS5A is a target for DAAs such as daclatasvir (DCV) that S225 can be one of the targets for such effective drugs. Goonawardane et al. [60] showed loss of S225 phosphorylation and DCV treatment resulted in NS5A structure changes, suggesting DAA treatment might target a function of NS5A. In addition, interference with NS5B phosphorylation sites completely inactivates this protein and lowers the replication efficiency of the subgenomic HCV replicons. Disruption of NS5A and NS5B phosphorylation suppresses HCV replication; hence, it has a detrimental effect on virion production and HCV pathogenesis. In the above-mentioned sentences, we have tried to shed light on the importance of NS5A and NS5B phosphorylation to help researchers to consider such possibilities in the production and manufacture of medicine to balance the therapeutic options by clinicians [61, 63].

The other protein post-modification process is glycosylation that are essential for viral assembly. Glycosylation can increase protein solubility and may be critical for maintaining NS5A functional structure [64–66]. The role of NS5B glycosylation was examined by Kanwal et al. [67] that found the number of N-linked glycosylation sites may contribute to the treatment outcome.

In our study, glycosylation prediction showed one site for 1a and three sites for 3a genotypes in which position 69 was similar in the two genotypes, which was in accordance with the report by Yamasaki et al. [64]. In 6 of the sample sequences, we found a mutation in amino acid 103, which caused omitting the glycosylation site in this position. NS5B glycosylation prediction showed only one position for 1a and two positions for genotype 3a without any similarity between the two genotypes. Although Kanwal et al. [67] used the same online software to predict glycosylation site for NS5B, they suggested completely different glycosylation sites that might be rooted in various virus strains.

Given the conservation of glycosylation, several compounds have been classified as carbohydrate-binding agents (CBAs) that show promise in the inhibition of virus activity. All in all, development of broad-spectrum antivirals to target glycosylation and selective deglycosylation can impair viral replication, assembly that offer an exciting prospect for several clinical treatments [68]. To the best of our knowledge, the effect of NS5 glycosylation proteins on HCV pathogenesis is not described clearly; hence, the experimental investigation is highly recommended to address this issue.

Prediction of disulfide bonds for NS5A sequences revealed several cysteines with significant similarities between the two genotypes. Six conserved cysteines (39, 59, 140, 142, 165, and 190) are common between the two genotypes, found in all patients’ sequences, which might be involved in NS5A function and structure.

Previous studies showed a C-terminal disulfide bond in the NS5A structure. The structure analysis revealed the presence of a novel fold, a zinc-coordination motif, and a C-terminal disulfide bond [3]. Consistent with our results, two cysteines (142 and 190) were found by Tellinghuisen et al. [69] confirming that these cysteines form a new zinc-coordination motif in NS5A domain I, which is vital for RNA replication. Lim et al. [70] suggested that the NS5A-NS5A interaction was required for NS5A function. Similar to our predictions, three cysteines (Cys-39, Cys-57, and Cys-59) were suggested for this interaction indicating the importance of these amino acids in NS5A function and structure. Ccysteines can form a zinc-binding motif which is vital for viral replication, NS5A binding to RNA, NS5A dimerization, and RNA binding. Due to the conserved positions of cysteines (Cys-39, Cys-57, Cys-59, Cys-142, and Cys-190) involved in the formation of disulfide bonds in HCV infected Iranian patients, it can be suggested such preserved positions may be involved in in HCV replication and liver disease progression. Consequently, disruption of disulfide bonds in the mentioned positions can be a promising therapeutic target to reduce viral activity.

As far as we know, there is a lack of published documents concerning NS5B disulfide bonds; however, our findings showed several disulfide bonds for NS5B in genotypes 1a and 3a. Since our data did not show any substitution in cysteine residues positions, these conserved sites might contribute to the fundamental activities associated with the host and HCV interactions.

All things considered, disulfide post modification analyses revealed that mutations may affect the number of bonds and bond patterns. Formation of the disulfide bond locks the structure in place and mostly increases the stability and half-life of the proteins [71]. Therefore, degradation of disulfide bonds can be a new area for development of antiviral drugs to degrade viral proteins.

NS5A B-cell epitope prediction showed some regions for the two genotypes, presenting the potential of this protein in inducing the humoral immune system. In comparison between the two genotypes, three regions (90–110, 180–197, and 215–233) were similar that can be beneficial in the new HCV vaccines development. In compliance with the present study, Ikram et al. [72] applied bioinformatics tools to select the most potent region to propose a novel vaccine. Despite different geographical regions, 2 regions of NS5A (185–190 and 220–225) were identical between the two studies, which confirms these immune-inducing regions have a great potential to be considered in vaccines against HCV infections.

Close to our prediction, Holmström et al. [73] indicated the potential of NS5A protein to induce high Ab levels, CTL responses, trigger CD8 + T cell triggering, and IFN-g induction, suggesting the ability of this protein to induce humoral and cellular immune systems. In our investigations, immunoinformatics analysis of NS5B revealed 2 B-cell epitopes for 1a and 3 regions for 3a genotypes, but there was no similar region between them. Latimer et al. [74] confirmed the potential of NS5B in inducing humoral and cellular immune systems. They designed a DNA vaccine consisting of more than 14 NS5B regions that two regions were close to our predictions (163–172 and 339–352). Noteworthy, our data showed these regions are highly conserved amongst different HCV genotypes. In addition, Kuhs et al. [75] used the same NS5B region to design a novel vaccine and confirmed the ability of this region to provoke the humeral immune system as well as triggering strong HCV-specific T cell responses in the spleen. In contrast to our bioinformatics analysis, one study considered another immune region of NS5A and NS5B proteins from HCV 2a/4a genotypes to induce humoral and persistent cellular responses in mice [76]. This dissimilarity might be related to the analysis of different HCV genotypes. All in all, combining our findings with previous ones confirm the ability of similar epitopes of NS5A and NS5B proteins to provoke the most intense humoral and cellular immune response for designing an effective vaccine construct.

Other than immune-bioinformatics analyses, protein interaction data, drug resistance-related mutation, etc. were other key factors that should be analyzed to characterize protein structure. Numerous studies used the SOPMA software to predict the secondary structure of different viral and bacterial proteins [77–79]. This software can predict 69.5% of amino acids for a three-state description of the secondary structure (alpha-helix, beta-sheet, and coil). In the present study, SOPMA showed that a large portion of NS5A and NS5B consists of a random coil and alpha helix. Several studies reported the majority structures of NS5A protein are coil [64] and helix [46, 64], which is in agreement with our data, and in some genotypes, B-Sheets were also defined [46, 64].

In our study, only epitopes located in random coil and β-turn structures were analyzed for antigenicity properties because such secondary structures are mostly located in the surfaces of the protein and are more easily to be recognized by antibodies. Different mutations did not affect the location of secondary structures and B cell epitopes [80]. Various post-translation modifications and B-cell epitope prediction suggested particular target sites and epitope regions for future antiretroviral drugs and vaccines design, respectively. Accordingly, NS5A and NS5B proteins have a great contribution in HCV replication; it seems the discrepancy between secondary structures in different HCV genotypes can influence virus pathogenicity. "I-TASSER" is the most famous program for predicting 3D structure of viral and bacterial proteins, reported by numerous studies that benefit from a hierarchical approach to protein structure and function prediction [81]. Using this software, we predicted the 3D structure of NS5A and NS5B proteins, and the proposed structures were refined by “GalaxyRefine”, and evaluation of the refined models showed the significant ability of this software in improving the quality and reliability of 3D models.

## Conclusion

Based on our findings, prescribing NS5A and NS5B inhibitors can effectively suppress HCV amongst Iranian HCV-infected patients. Despite some substitutions in NS5A and NS5B proteins, such minor mutations would not significantly affect the efficiency of the NS5 inhibitors. In addition, the suggested B cell epitopes in NS5A and NS5B proteins can remarkably trigger an immune response that can be profitable in HCV vaccine production. As phosphorylation and glycosylation are essential to form active NS5A and NS5B proteins, the yeast host not only may support PMTs but also can increase the protein expression yield. Moreover, NS5A and NS5B post-modifications including phosphorylation, glycosylation, and formation of disulfide bonds are crucial for HCV pathogenesis. PTM pathways and PTM sites are potential pharmacological targets for new therapeutic approaches. To unveil the precise PTM sites and the underlying mechanisms much work remains to be done.

## Supplementary Information

**Additional file 1**. Figs. S1-S12 illustrated the amino acids involved in docking analysis between NS5A and B with NS5 inhibitors including Daclatasvir, Elbasvir, Ledipasvir, and Ombitasvir.

## Data Availability

Data that support the findings of this study are available from the corresponding author (Dr. Ava Hashempour) upon reasonable request.
