# Supplementary material for: First report on molecular docking analysis and drug resistance substitutions to approved HCV NS5A and NS5B inhibitors amongst Iranian patients
Source: BMC Gastroenterol. 2021 Nov 24;21:443. doi: 10.1186/s12876-021-01988-y (PMC8612383; doi:10.1186/s12876-021-01988-y)

**S1.** Docking analysis between NS5A 1a and [Daclatasvir](https://en.wikipedia.org/wiki/Daclatasvir)


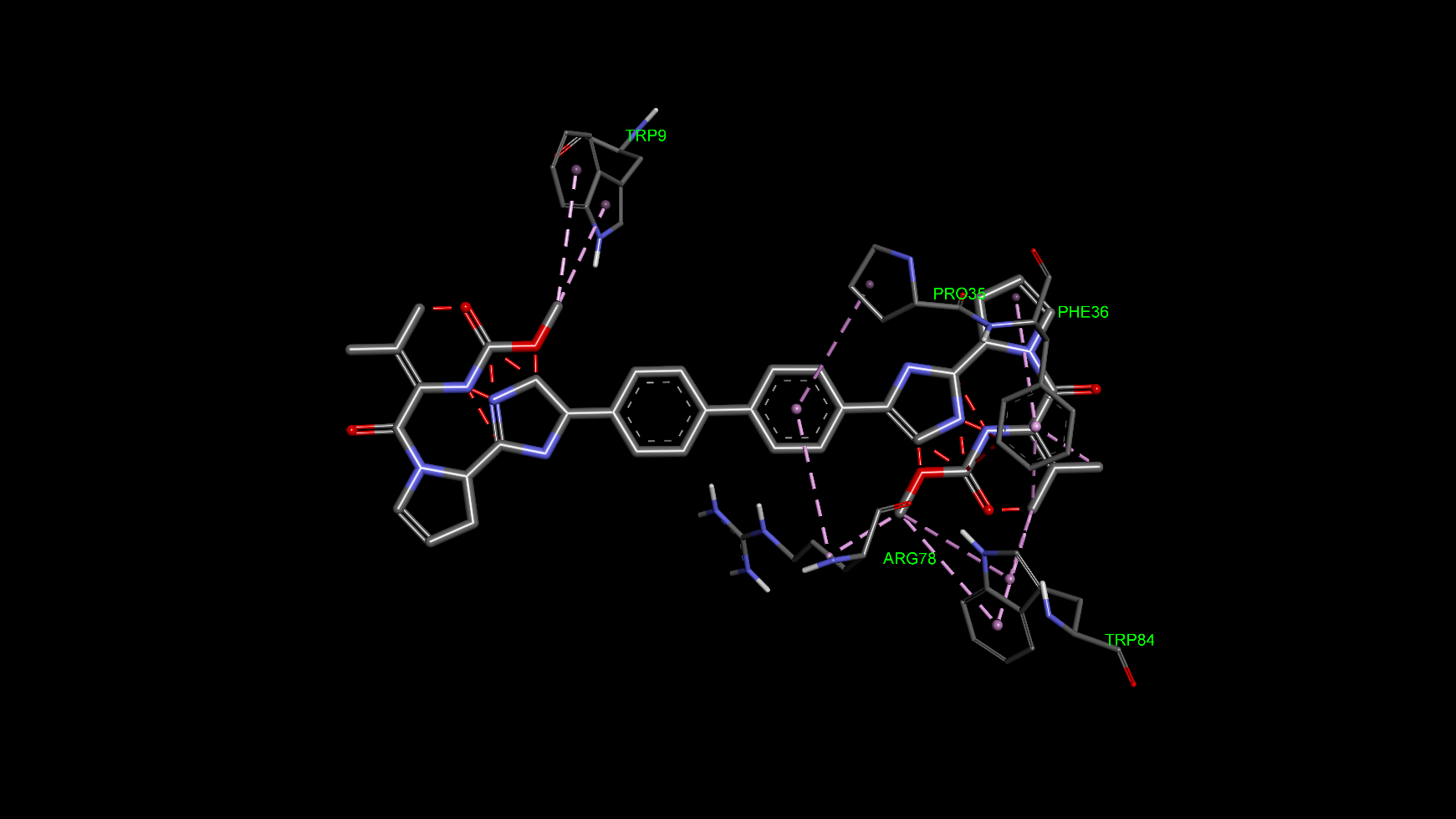


**S2.** Docking analysis between NS5A 1a and [Elbasvir](https://en.wikipedia.org/wiki/Daclatasvir)


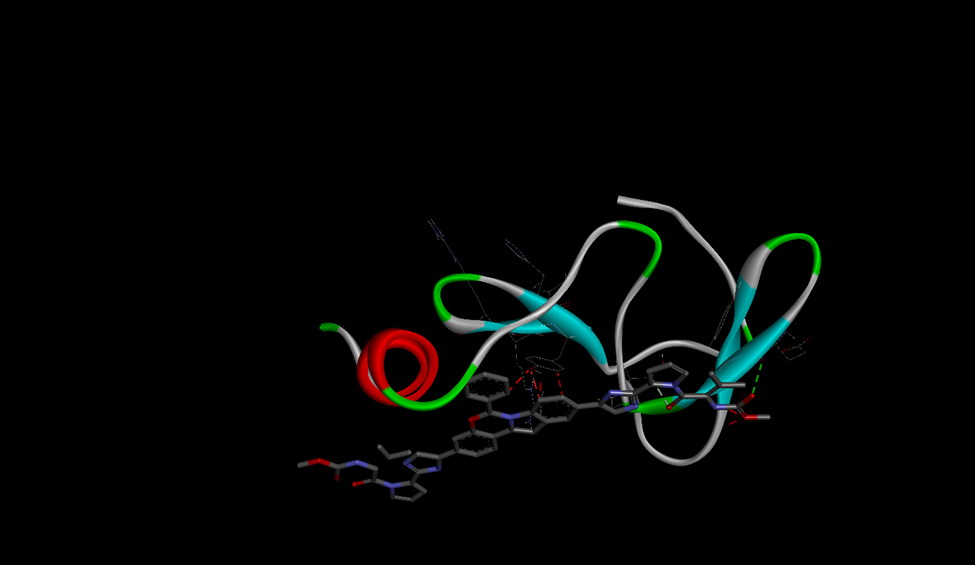


**S3.** Docking analysis between NS5A 1a and [Ledipasvir](https://en.wikipedia.org/wiki/Daclatasvir)


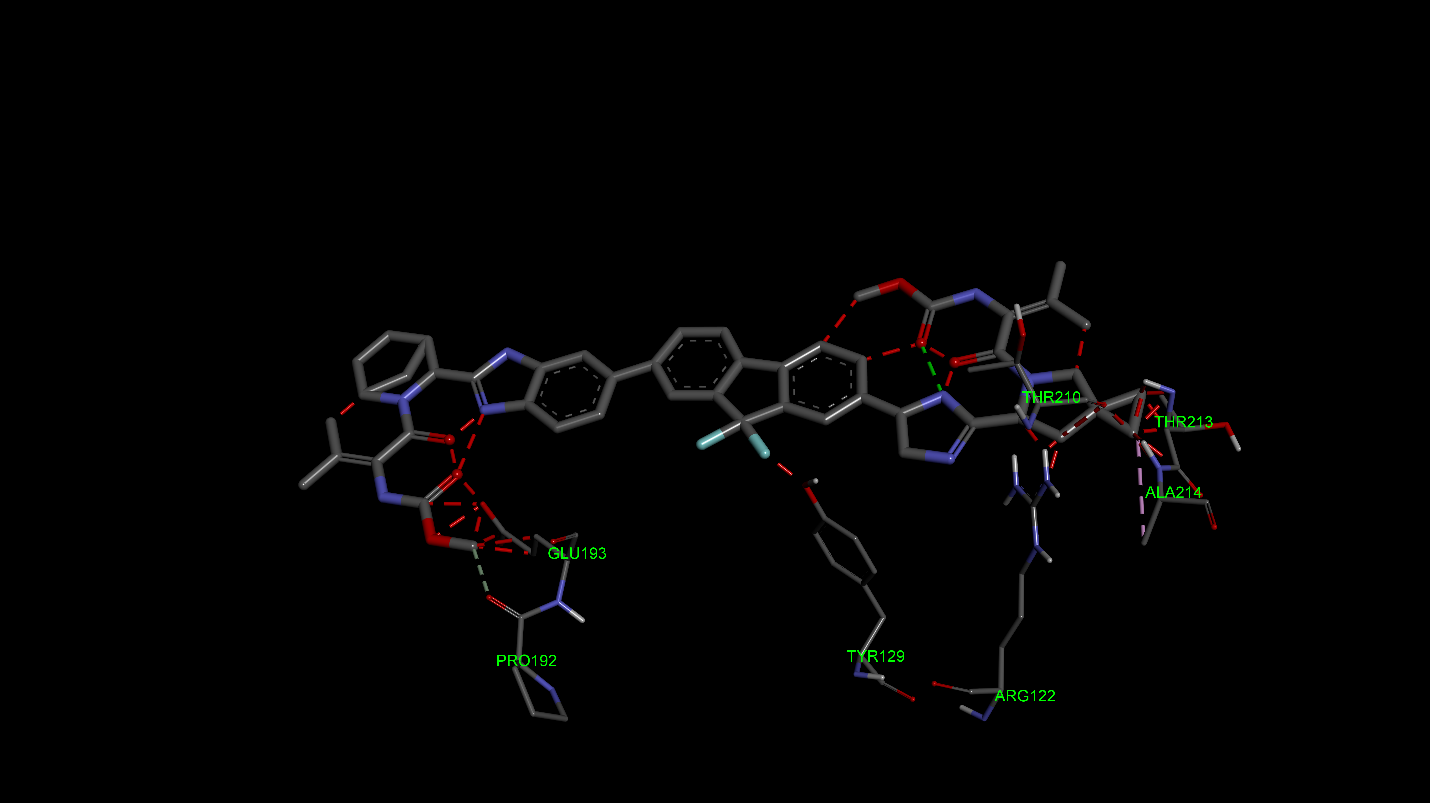


**S4.** Docking analysis between NS5A 1a and [Ombitasvir](https://en.wikipedia.org/wiki/Daclatasvir)


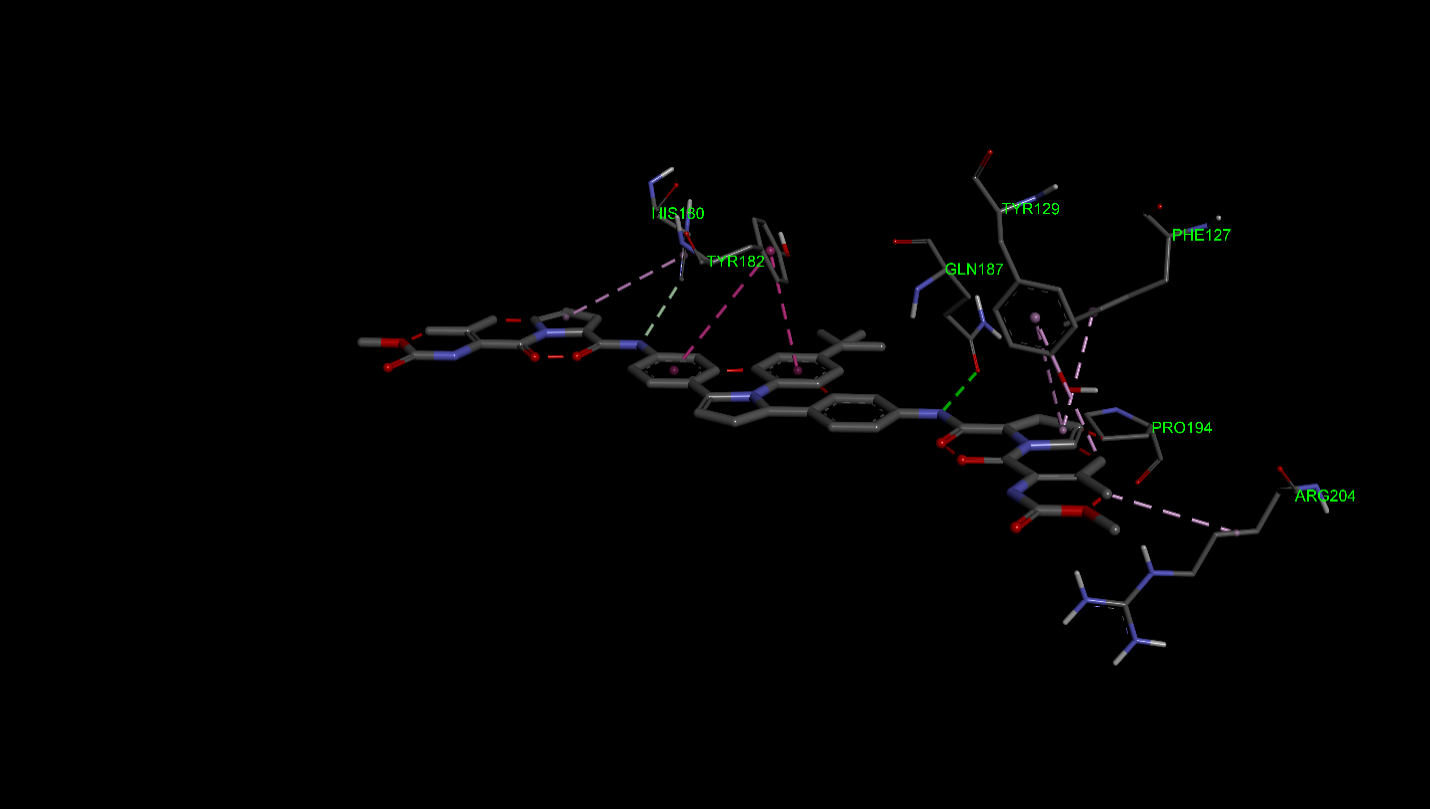


**S5.** Docking analysis between NS5A 3a and [Daclatasvir](https://en.wikipedia.org/wiki/Daclatasvir)


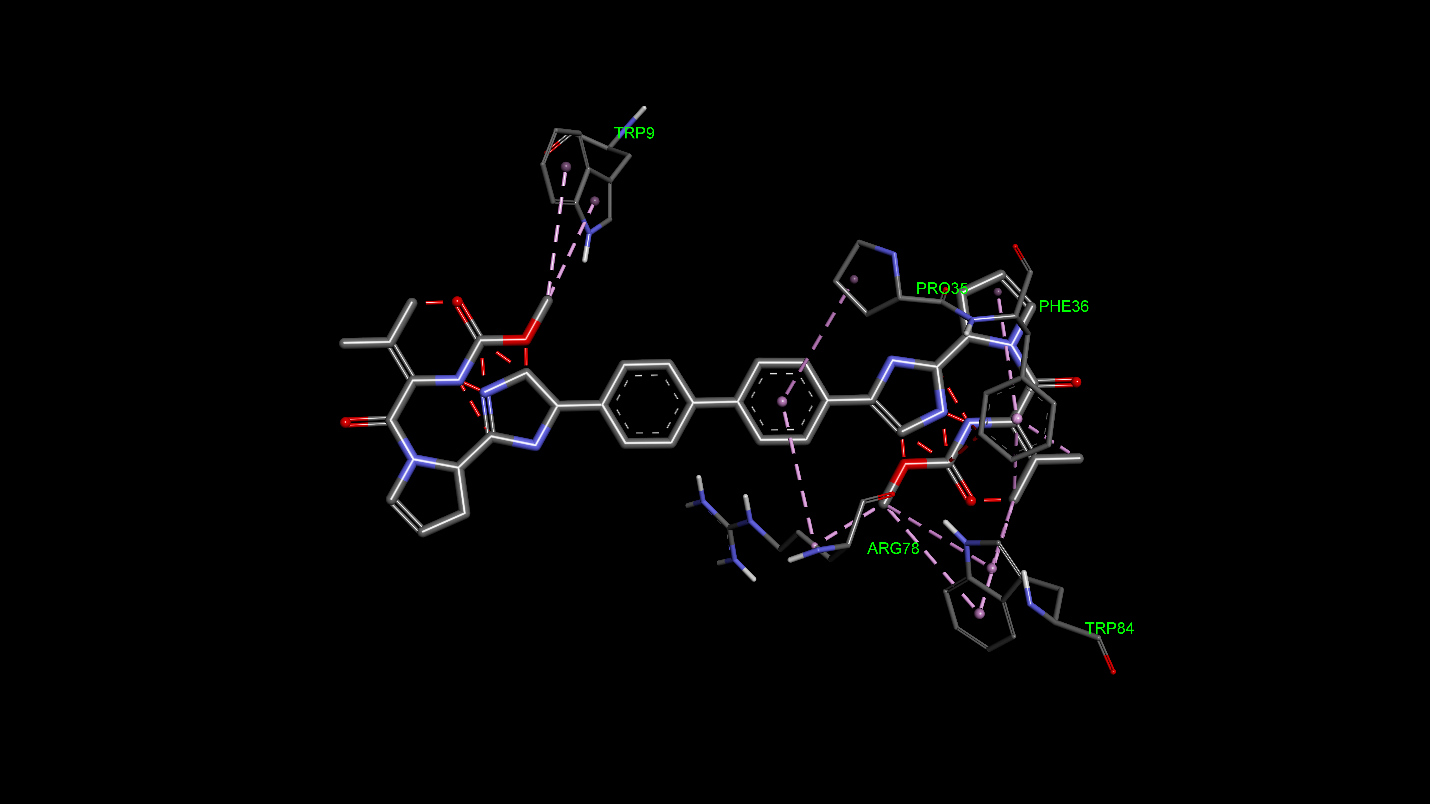


**S6.** Docking analysis between NS5A 3a and [Elbasvir](https://en.wikipedia.org/wiki/Daclatasvir)


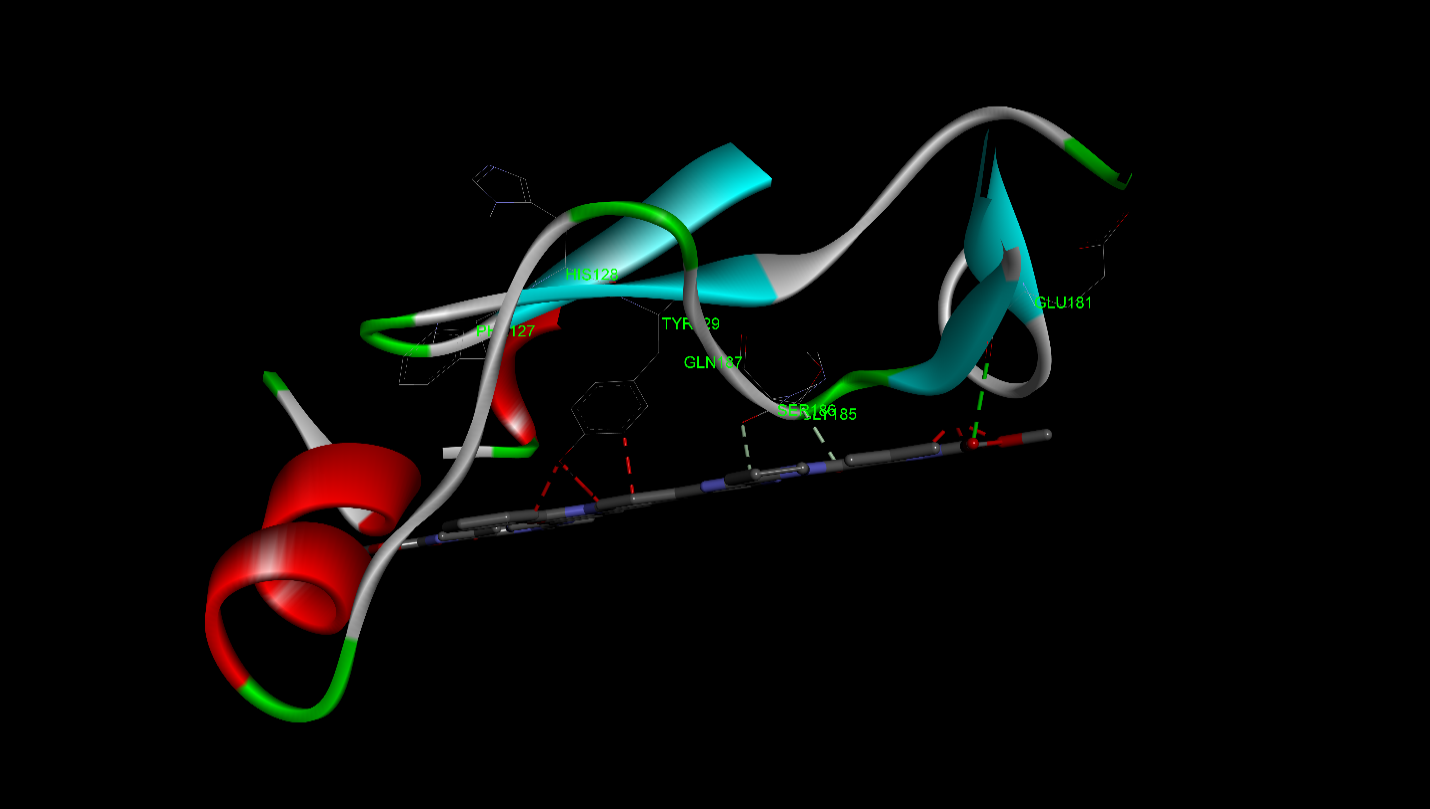


**S7.** Docking analysis between NS5A 3a and [Ledipasvir](https://en.wikipedia.org/wiki/Daclatasvir)


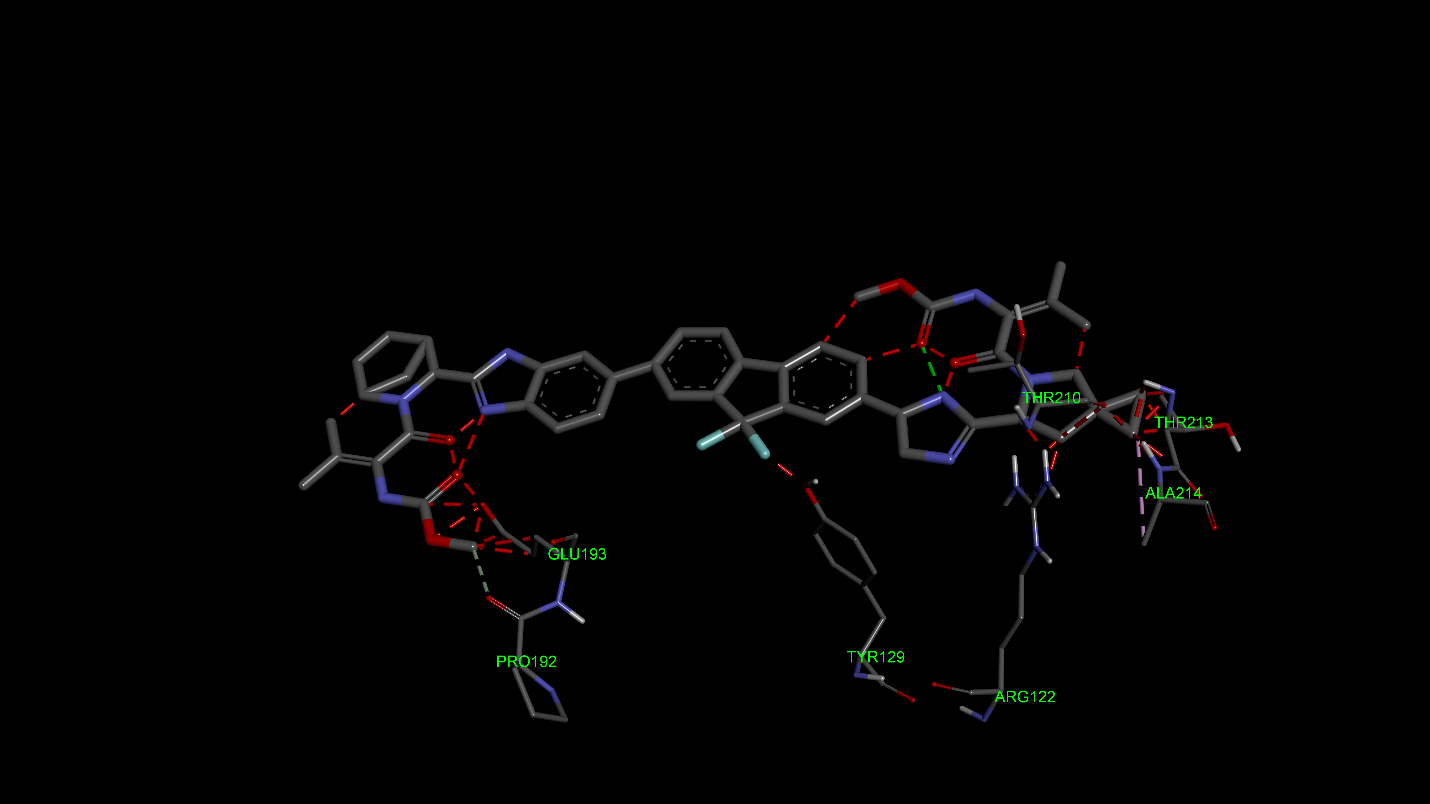


**S8.** Docking analysis between NS5A 3a and [Ombitasvir](https://en.wikipedia.org/wiki/Daclatasvir)


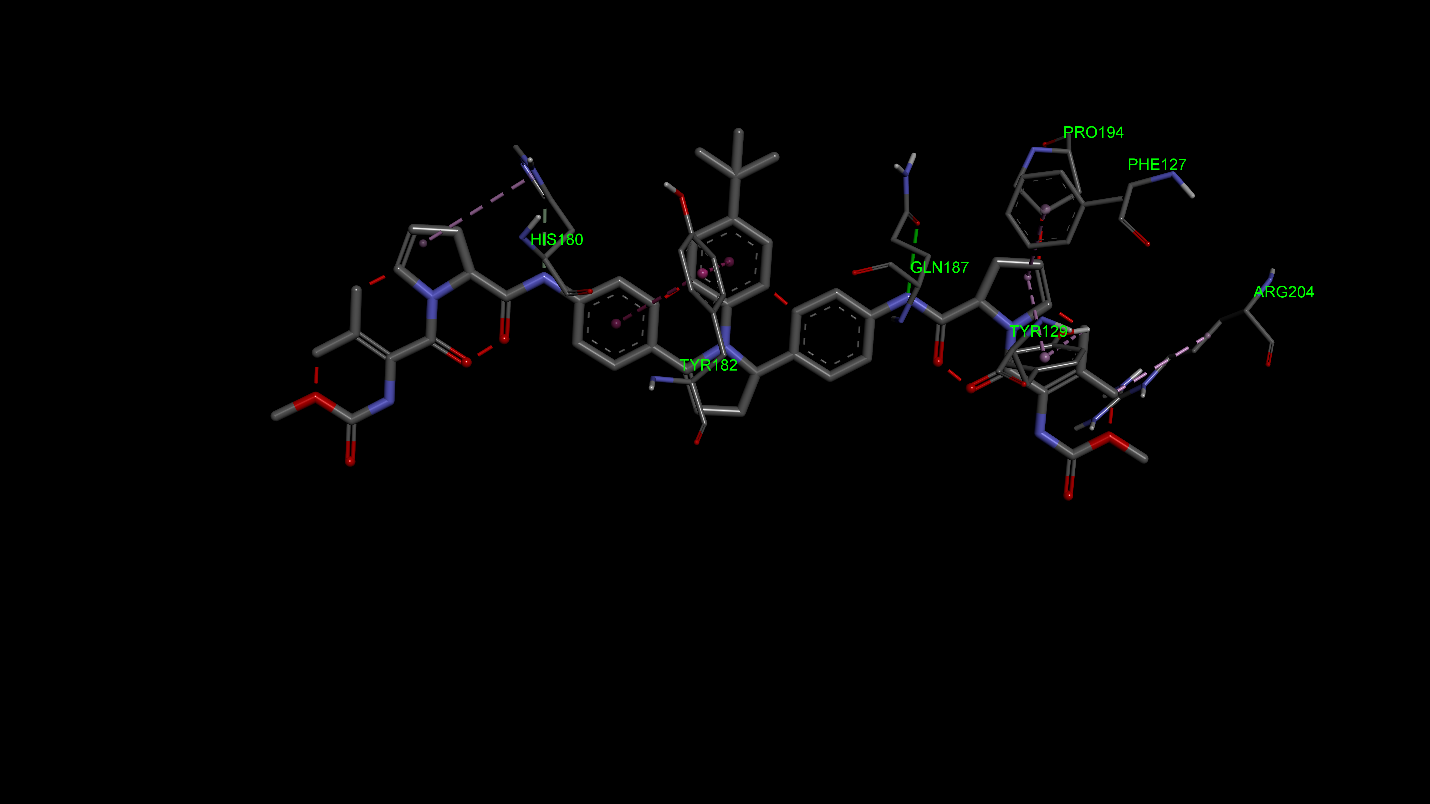


**S9.** Docking analysis between NS5B 1a and [Dasabuvir](https://en.wikipedia.org/wiki/Daclatasvir)


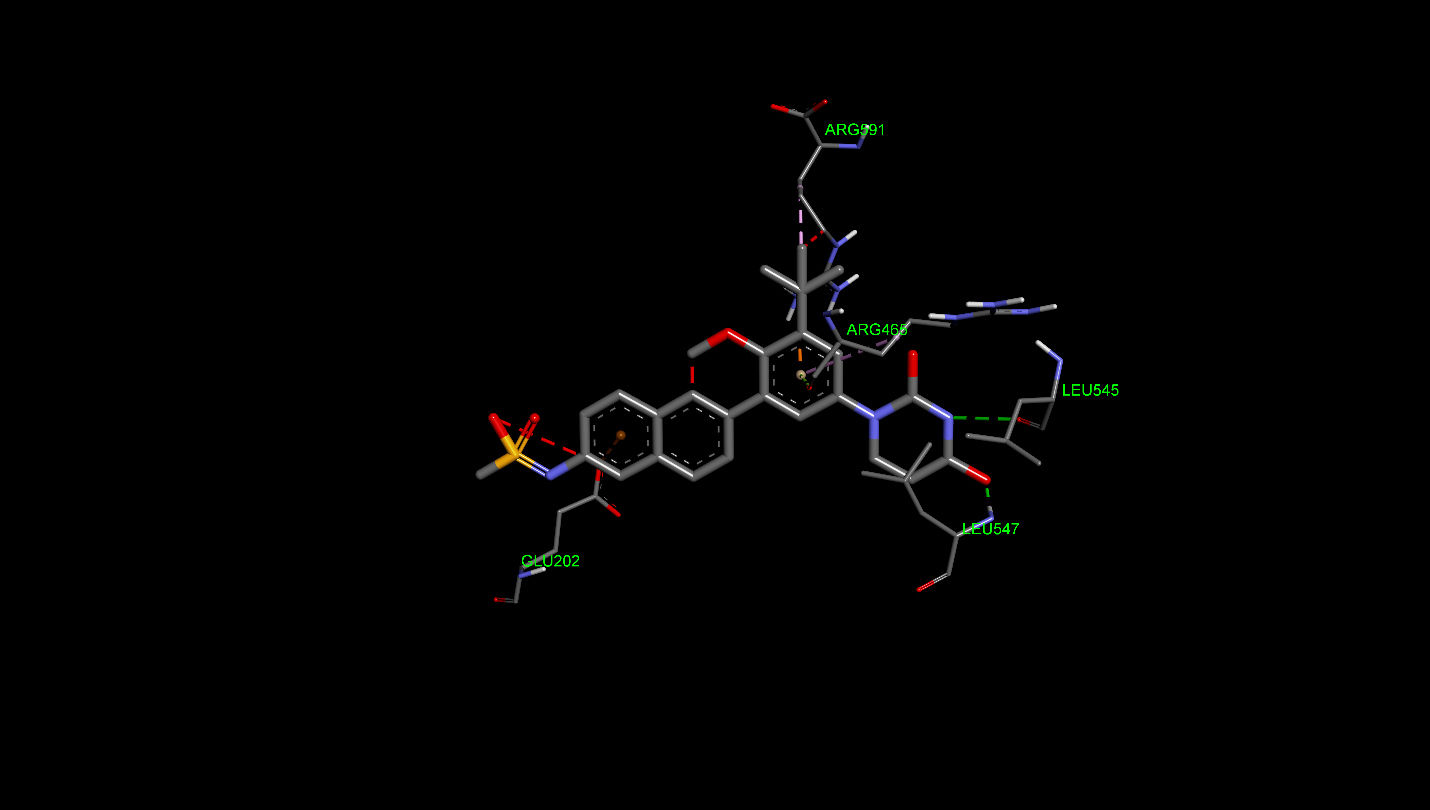


**S10.** Docking analysis between NS5B 1a and [Sofosbuvir](https://en.wikipedia.org/wiki/Daclatasvir)


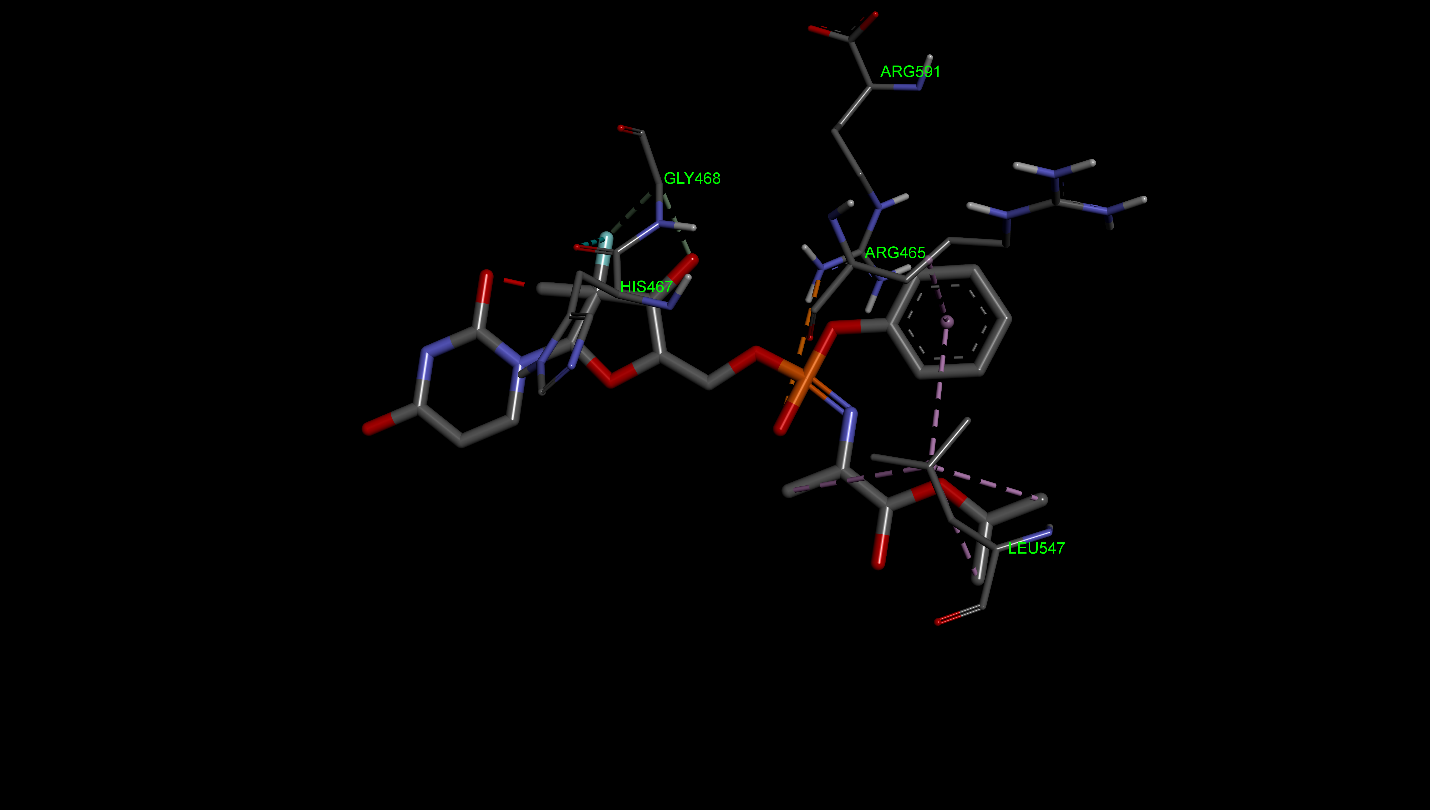


**S11.** Docking analysis between NS5B 3a and [Dasabuvir](https://en.wikipedia.org/wiki/Daclatasvir)


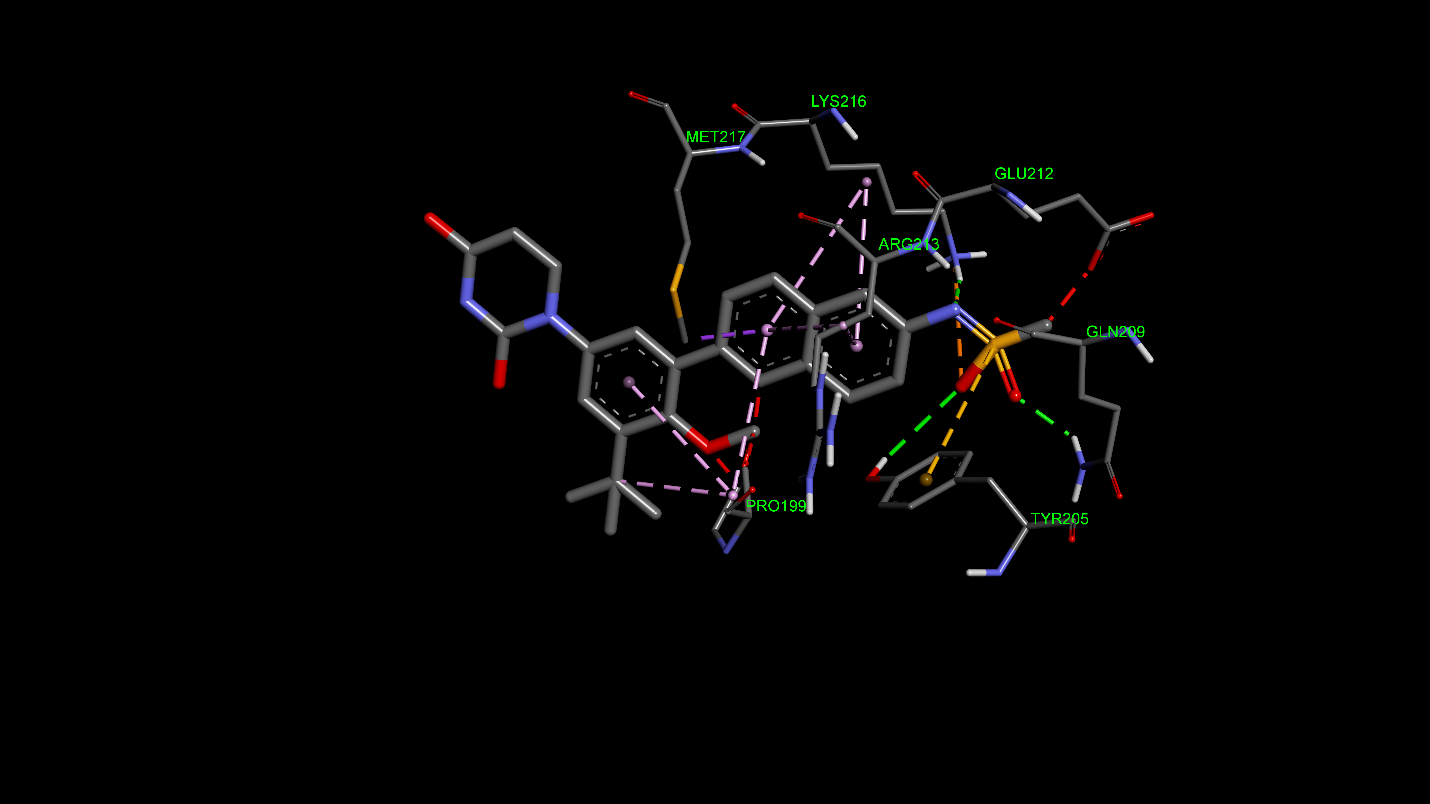


**S12.** Docking analysis between NS5B 3a and [Sofosbuvir](https://en.wikipedia.org/wiki/Daclatasvir)


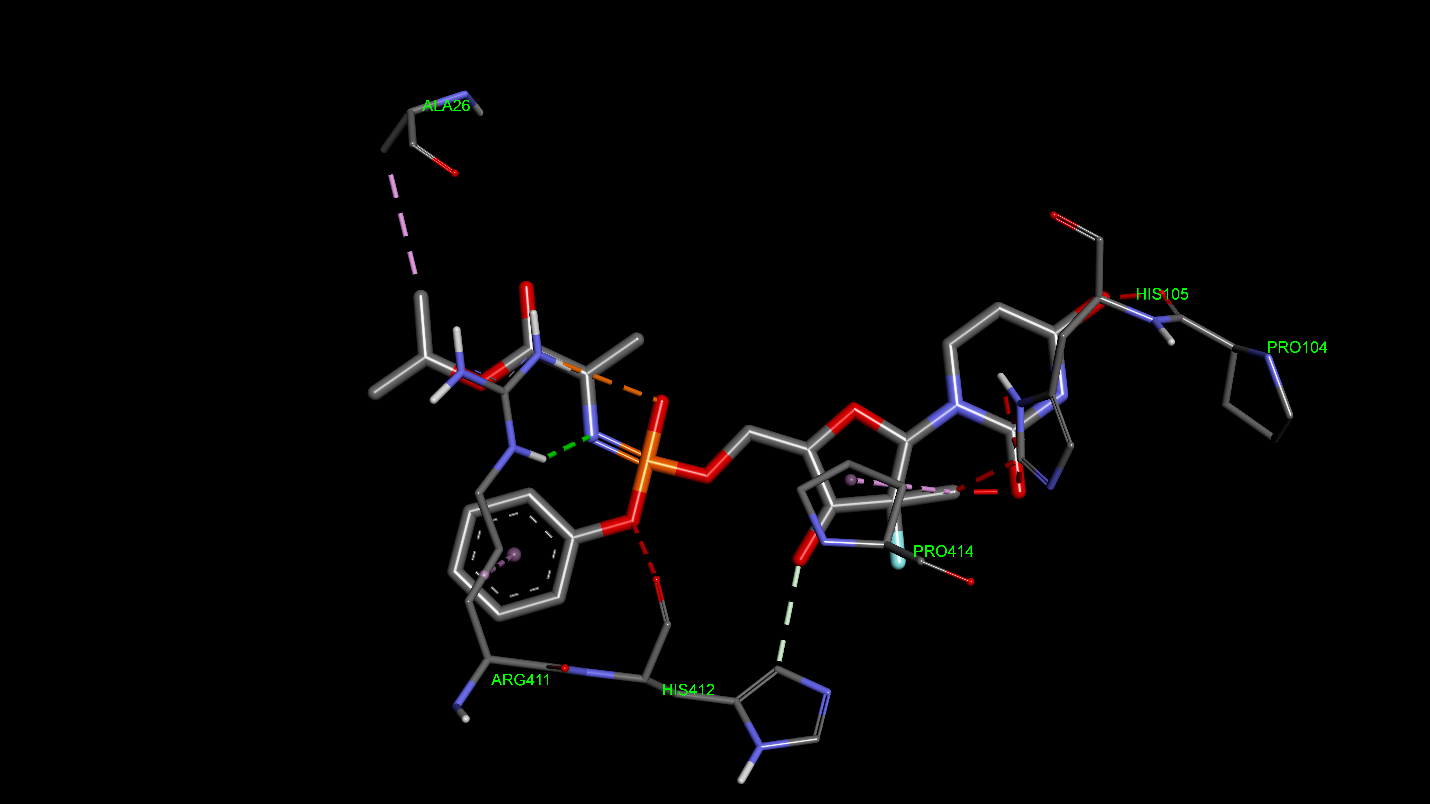

Supplement: Supplementary file 1 — Additional file 1. Figs. S1-S12 illustrated the amino acids involved in docking analysis between NS5A and B with NS5 inhibitors including Daclatasvir, Elbasvir, Ledipasvir, and Ombitasvir. [file 12876_2021_1988_MOESM1_ESM.docx]
